# Perceptual Weighting of Binaural Lateralization Cues across Frequency Bands

**DOI:** 10.1007/s10162-020-00770-3

**Published:** 2020-09-11

**Authors:** Axel Ahrens, Suyash Narendra Joshi, Bastian Epp

**Affiliations:** 1 Hearing Systems Section, Department of Health Technology, Technical University of Denmark, Kgs. Lyngby, Denmark; 2 Present Address: Brain & Sound Lab, Department of Biomedicine, Basel University, Basel, Switzerland

**Keywords:** binaural hearing, spectral integration, spectral weighting, auditory enhancement, 43.64.-q, 43.64.+r, 87.19.lt, 43.66.-x, 43.66.+y

## Abstract

The auditory system uses interaural time and level differences (ITD and ILD) as cues to localize and lateralize sounds. The availability of ITDs and ILDs in the auditory system is limited by neural phase-locking and by the head size, respectively. Although the frequency-specific limitations are well known, the relative contribution of ITDs and ILDs in individual frequency bands in broadband stimuli is unknown. To determine these relative contributions, or spectral weights, listeners were asked to lateralize stimuli consisting of eleven simultaneously presented 1-ERB-wide noise bands centered between 442 and 5544 Hz and separated by 1-ERB-wide gaps. Either ITDs or ILDs were varied independently across each noise band, while fixing the other interaural disparity to either 0 dB or 0 μs. The weights were obtained using a multiple linear regression analysis. In a second experiment, the effect of auditory enhancement on the spectral weights was investigated. The enhancement of single noise bands was realized by presenting ten of the noise bands as preceding and following sounds (pre- and post-cursors, respectively). Listeners were asked to lateralize the stimuli as in the first experiment. Results show that in the absence of pre- and post-cursors, only the lowest or highest frequency band received highest weight for ITD and ILD, respectively. Auditory enhancement led to significantly enhanced weights given to the band without the pre- and post-cursor. The weight enhancement could only be observed at low frequencies, when determined with ITD cues and for low and high frequencies for ILDs. Hence, the auditory system seems to be able to change the spectral weighting of binaural information depending on the information content.

## **INTRODUCTION**

An important ability of the auditory system is spatial hearing. This ability enables the localization of sound sources in auditory space (see Middlebrooks and Green 1991, for a review) and to improve the understanding of speech in environments with interfering sound sources (e.g., Bronkhorst 2000). The complete set of cues underlying spatial hearing can be derived from the head-related transfer functions of the two ears relative to the location of the sound source. Two of these cues are interaural disparities in time (interaural time difference, ITD) and interaural disparities in level (interaural level difference, ILD). When presented in isolation under headphone conditions, sounds with ITD and ILD information are commonly not localized in space outside the listeners’ head but rather lateralized towards one side inside the listeners’ head. Both ITDs and ILDs separately as well as in combination allow a listener to lateralize an acoustic stimulus. Under free-field conditions, ITDs are predominantly used at low frequencies and ILDs are predominantly used at high frequencies (Rayleigh 1907). The spectral dominance of ITD and ILD is commonly referred to as the Duplex theory and are widely acknowledged in the literature (e.g., Macpherson and Middlebrooks 2002). It is, however, less clear what the contribution of the different cues in the different frequency bands is in more realistic conditions with broadband stimuli.

At low frequencies, the ITD is a reliable cue and small changes in ITD can be detected. At high frequencies, the ITD of the fine structure becomes imperceptible (Rayleigh 1907; Blauert 1984; Moore 2014; Brughera et al. 2013) and hence unavailable as a cue for lateralization. For narrow band signals, ITD detection thresholds have been shown to be lowest between 700 and 1000 Hz and to increase towards lower and higher frequencies (Klumpp and Eady 1956; Brughera et al. 2013). The upper frequency limit of fine structure ITD detection was shown to be at about 1.5 kHz (Moore 2014; Zwislocki and Feldman 1956; Klumpp and Eady 1956; Brughera et al. 2013). At frequencies above 1.5 kHz, ITD cues in the envelope of a signal can be detected when imposed on carrier frequencies well above 1.5 kHz (Henning 1974; McFadden and Pasanen 1976; Bernstein and Trahiotis 1994; Nuetzel and Hafter 1976; Leakey et al. 1958). At high frequencies, the wavelength of the sound waves becomes small in comparison with the size of the head. This leads to a reduction in sound intensity at the contralateral ear relative to the ipsilateral ear and leads to ILD cues (Blauert 1984). ILD detection thresholds have been shown to be approximately constant over a broad range of frequencies, except at 1 kHz where the threshold has been shown to be higher (Yost and Dye 1988; Grantham 1984; Rowland and Tobias 1967; Mills 1960).

Previous studies have suggested that the frequency-specific detection thresholds of the interaural disparities are related to their relative contribution to spatial hearing. One approach used to derive the spectral weighting is to invert discrimination thresholds for narrowband signals and to calculate sensitivity under the assumption that high sensitivity indicates a high relative contribution, and hence a high weighting for the cue in that frequency band. For ILDs, this approach leads to a constant ILD weighting across frequency bands and for ITDs to a weighting that is maximal between 700 and 1000 Hz and decreases towards high and low frequencies (Raatgever 1980; Stern et al. 1988; Buchholz et al. 2018).

However, detection (McFadden and Pasanen 1976) and discrimination (Heller and Richards 2010; Trahiotis and Bernstein 1990) thresholds of ITD and ILD as well as the lateralization extent (Heller and Trahiotis 1996) are affected by the presence of signals in remote spectral regions, a phenomenon known as binaural interference. For example, the ITD threshold of a probe in a frequency band is increased in the presence of an interfering signal in a frequency band lower than the probe frequency (Best et al. 2007). Thus, the spectral weights obtained by inverting thresholds for narrowband signals in isolation might not be applicable for broadband signals.

Furthermore, Buell and Hafter (1991) showed that binaural information are summed across frequency bands only if the information belong to the same auditory object. Thus, when multiple auditory objects of target and interfering signal are formed, no binaural interference occurs. In addition, it has been shown that pre- and post-cursors, i.e., signals preceding and following a target, can reduce the detection threshold of a masked signal and make the signal perceptually ‘pop out’ of a simultaneous interfering signal, also referred to as auditory enhancement (Viemeister 1980). Byrne et al. (2011) showed that such an enhancement paradigm led to a 4–5 dB increase in perceived level of the target signal in a binaural centering task. Thus, the effect of auditory enhancement might also affect the apparent spectral weighting of the interaural disparities.

In the present study, we investigated how the auditory system integrates either of the binaural cues of ITD or ILD across frequency for broadband signals in a lateralization task. An observer-response weighting analysis paradigm was used to determine relative contributions of spectral bands to sound lateralization. Previously, this analysis has been used to estimate the spectral and temporal weights for the judgment of spectral shape (Lutfi and Jesteadt 2006; Berg 1990), for spectral weights of loudness (Jesteadt et al. 2014; Joshi et al. 2016; Leibold et al. 2007; Leibold et al. 2009; Oberfeld et al. 2012), for level discrimination (Lutfi 1989; Kortekaas et al. 2003), and for temporal weights of ITDs and ILDs (Stecker and Hafter 2002; Brown and Stecker 2010; Brown and Stecker 2011; Stecker et al. 2013; Stecker 2014; Stecker 2018; Dye et al. 2005). Using an observer-response weighting analysis enables the estimation of weights using stimuli with interaural disparities above threshold and taking binaural interference into consideration. In the first experiment, the spectral weights were derived by imposing semi-random permutations of ITD or ILD in multiple frequency bands and asking listeners to lateralize (left or right) the stimulus. This was done separately for ITD and ILD. In a second experiment, the spectral weights of ITDs and ILDs were derived in the presence of pre- and post-cursors to investigate the effect of auditory enhancement on the spectral weights. The range of values of the ITDs and ILD were chosen to be independent of frequency to anticipate potential differences in ITD and ILD detection threshold between the stimulus used in the current study and isolated pure tones or narrow band noises as previously used in literature.

## **METHODS**

### Experiment 1: Spectral Weights in Static Condition

**Fig. 1:**
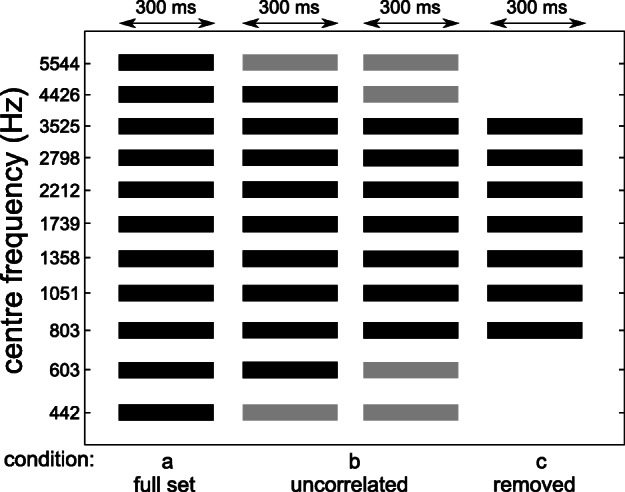
Schematic of the experimental conditions used in experiment 1. The black bars indicate noise bands with interaural disparities and the gray bars indicate bands with interaurally uncorrelated noise. All bands had a spectral width of 1-ERB and were spaced 1-ERB from each other. All bands had a duration of 300 ms

Figure 1 shows a schematic of the stimuli used in experiment 1. Each bar indicates a 1-ERB (Equivalent Rectangular Bandwidth, Moore 1983; Glasberg and Moore 1990)-wide noise band. For each trial, a Gaussian white noise was filtered into 11 noise bands using 4th order gammatone filters at the desired center frequencies separated by 1-ERB-wide spectral gaps. In this study, 11 ITD or ILD values were used (see details below). The 11 values of the interaural disparities were permuted and applied to the noise bands. Each permutation resulted in a block of 11 stimuli making one trial. The 11 permutations resulted in 11 trials with every interaural disparity occurring once per noise band and in total 121 stimuli per run. Each of the runs was repeated five times with a randomized order of the 121 stimuli. For each run, the same noise instances were used for all repetitions and listeners. Furthermore, the same permutations of the binaural disparities across the frequency bands were presented to the listeners but in different random orders.

The duration of the noise bands was 300 ms with 2-ms on-/offset ramps (raised-cosine window). The sampling frequency was 48 kHz. To obtain ITDs, the waveform of each noise band was shifted by the specific ITD value in one of the headphone channels. ILDs were achieved by raising the sound level of a given noise band at one ear by half of the desired ILD and lowering the level at the other ear by an equal amount.

In experiment 1a, the center frequencies of the noise bands ranged from 442 to 5544 Hz. In one condition, ILDs were held constant at 0 dB and 11 ITD values ranging from − 500 to + 500 μs in steps of 100 μs were applied pseudo-randomly to the 11 noise bands. In the other condition, ITDs were held constant at 0 μs and 11 ILD values from − 5 to + 5 dB in steps of 1 dB were applied. Negative values indicate a leading left ear and positive values a leading right ear.

In experiment 1b, the most outer or the two most outer noise bands (referred to as the “edge bands”) were presented as interaurally uncorrelated noises. Thus, these frequency bands contained no useful information for lateralization. The light gray bars in Fig. 1 indicate the bands with binaurally uncorrelated noise, while the dark bars indicate the bands with fully correlated noise and interaural disparities. In experiment 1c, the two most outer bands were removed.

The stimuli were presented over headphones (Sennheiser HDA200; Sennheiser Electronic GmbH & Co. KG, Wedemark, Germany) with an overall level of 60 dB SPL. The headphones were equalized with a 2048 point FIR filter modeling the inverted headphone transfer function measured on a B&K 4153 artificial ear (Brüel&Kjær Sound&Vibration Measurement A/S, Nærum, Denmark). The subjects were placed in a single-walled soundproof booth. After each stimulus, they were asked to indicate if the sound was perceived as coming from the left or from the right side (1-interval, 2-alternative forced choice). No feedback was provided. A single run took the subjects about 3 to 4 min and breaks were allowed after every run. Sessions lasted a maximum of 2 h.

To derive the spectral weights for the interaural disparities, a model was assumed where the physical input data (ITD or ILD values over center frequencies) were mapped to the psychological output data (subject responses). The input variable (interaural disparities) was scaled to cover a range between −1 and +1. The frequency-dependent mapping variable was the weight that the listener put on a particular spectral region during the decision. The mapping variable (or spectral weight) was determined by using a multiple linear regression analysis between the interaural disparities and the listener’s response. The multiple linear regression analysis was performed using the MATLAB (The MathWorks Inc., Natick, Massachusetts, USA) function *fitlm()* which returns the weights. A weight of zero would reflect no impact of the corresponding frequency band on the lateralization.

### Experiment 2: Spectral Weights with Auditory Enhancement

**Fig. 2:**
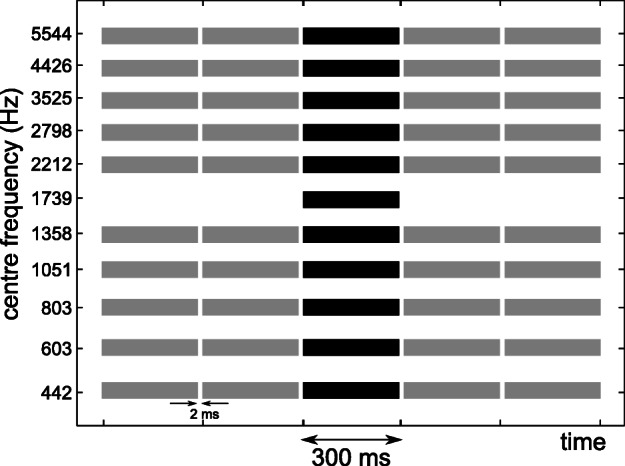
Schematic of one of the stimuli used in experiment 2. The black bars indicate noise bands with interaural disparities and the gray bars indicate the pre- and post-cursors. The pre- and post-cursor bands contained diotic noise. All bands had a spectral width of 1-ERB and were spaced 1-ERB from each other. All bands had a duration of 300 ms with 2-ms gaps between bands. In this example, the on-frequency band was the 1739-Hz band. The remaining bands are referred to as off-frequency bands. On-frequency bands at 442, 803, 3525, and 5544 Hz were also tested

In experiment 2, a paradigm inspired by auditory enhancement experiments was adopted to investigate the effect of spectro-temporal context on the spectral weights of ITD and ILD. The enhancement of a specific frequency band (referred to as on-frequency band) was promoted by using pre- and post-cursors, which were only applied to the off-frequency bands. Figure 2 shows an example schematic of the stimulus. The procedures to create the target (black bars) and to determine the spectral weights were the same as in experiment 1. The pre-and post-cursors (gray bars) were diotic noise bands with a bandwidth of 1-ERB and a duration of 300 ms. Two noise intervals were presented before and after each off-frequency band. A gap of 2 ms was introduced between each interval and between the pre- and post-cursor and the target. Five on-frequency bands were tested separately: 442, 803, 1739, 3525, and 5544 Hz.

### Listeners

Ten listeners (6 female, 4 male) participated in experiment 1 and a subset of 6 listeners in experiment 2. The listeners were paid on an hourly basis. The hearing thresholds of the listeners measured prior to the experiment showed audiometrically normal hearing (< 20 dB HL between 125 Hz and 8 kHz). The participants were between 24 and 30 years old (average 25.2 years). Four of the listeners had previous experience with psychoacoustic experiments. All participants provided informed consent and all experiments were approved by the Science-Ethics Committee for the Capital Region of Denmark (reference H-KA-04149-g).

### Statistical Analyses

The statistical analyses were performed using the statistical computing software R (version 3.6.1). Linear mixed effects models were fitted to either of the interaural disparities using the *lmerTest* package (Kuznetsova et al. 2014). The effect of the listeners was treated as a random factor. If within factor comparisons were performed, the *emmeans* package (Lenth 2016) was used with the Satterthwaite method to calculate the degrees of freedom. The post hoc *p* values were corrected for multiple comparisons using the Bonferroni correction. In the plots, asterisks and dots are used to indicate the *p* values. Three asterisks indicate a *p* value smaller than 0.001, two asterisks smaller than 0.01, a single asterisk smaller than 0.05 and a dot a *p* value between 0.05 and 0.1. If no asterisk is plotted in the figure, no significant difference was found.

## **RESULTS**

### Experiment 1: Spectral Weights in Static Condition

#### Spectral Weights

**Fig. 3:**
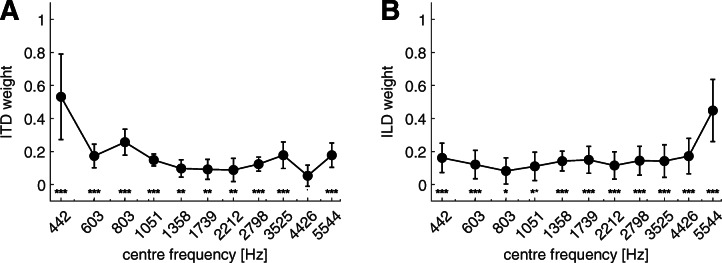
Spectral weights of ITDs (panel A) and ILDs (panel B) with respect to the center frequencies of the 1-ERB noise bands. The circles and the error bars indicate the mean and the standard deviation for the ten listeners, respectively. The results from the post hoc analysis across frequency bands are shown in Tables 1 and 2. The asterisks indicate *p* values of weights relative to zero. ***p*** < ***0.0001, **p*** < ***0.01, *p ***< ***0.05, ·*p* ***< ***0.1

Figure 3 shows the spectral weights for ITDs (panel A, left) and ILDs (panel B, right) with respect to the center frequencies of the noise bands. The analysis of a linear mixed model showed a significant effect of the frequency bands for both ITD [*F*(10,90) = 19.77, *p* < 0.0001] and ILD [*F*(10,99) = 16.58, *p* < 0.0001]. The asterisks in Fig. 3 indicate the significance of each weight with respect to 0. The statistical values of the comparisons across the frequency bands are shown in Tables 1 and 2 for the spectral weights of ITDs and ILDs, respectively. *p* values in the Tables with a value ≤ 0.05 are indicated in italics.

**Table 1:** The *t*-ratio (bottom, left) and the corrected *p* values (top, right) from the multiple comparison analysis of the spectral weights for the ITDs

p value	442 Hz	603 Hz	803 Hz	1051 Hz	1358 Hz	1739 Hz	2212 Hz	2798 Hz	3525 Hz	4426 Hz	5544 Hz
*t*-ratio											
442 Hz	-	*< 0.0001*	*< 0.0001*	*< 0.0001*	*< 0.0001*	*< 0.0001*	*< 0.0001*	*< 0.0001*	*< 0.0001*	*< 0.0001*	*< 0.0001*
603 Hz	*8.58*	-	1.0	1.0	1.0	1.0	1.0	1.0	1.0	0.28	1.0
803 Hz	*6.58*	− 2.0	-	0.62	*0.0135*	*0.0086*	*0.0063*	0.11	1.0	*0.0003*	1.0
1051 Hz	*9.17*	0.58	2.59	-	1.0	1.0	1.0	1.0	1.0	1.0	1.0
1358 Hz	*10.4*	1.82	*3.82*	1.23	-	1.0	1.0	1.0	1.0	1.0	1.0
1739 Hz	*10.52*	1.94	*3.95*	1.36	0.13	-	1.0	1.0	1.0	1.0	1.0
2212 Hz	*10.61*	2.03	*4.04*	1.45	0.22	0.09	-	1.0	1.0	1.0	1.0
2798 Hz	*9.8*	1.18	3.19	0.6	− 0.63	− 0.76	− 0.85	-	1.0	1.0	1.0
3525 Hz	*8.47*	− 0.11	1.89	− 0.69	− 1.93	− 2.05	− 2.14	− 1.29	-	0.2	1.0
4426 Hz	*11.45*	2.87	*4.88*	2.29	1.06	0.93	0.84	1.69	2.98	-	0.2
5544 Hz	*8.47*	− 0.11	1.89	− 0.7	− 1.93	− 2.05	− 2.14	− 1.29	0.0	− 2.98	-

The degrees of freedom are 99. Combinations with a *p* value smaller 0.05 are indicated in italics

**Table 2:** The *t*-ratio (bottom, left) and the corrected *p* values (top, right) from the multiple comparison analysis of the spectral weights for the ILDs

p value	442 Hz	603 Hz	803 Hz	1051 Hz	1358 Hz	1739 Hz	2212 Hz	2798 Hz	3525 Hz	4426 Hz	5544 Hz
*t*-ratio											
442 Hz	-	1.0	1.0	1.0	1.0	1.0	1.0	1.0	1.0	1.0	*< 0.0001*
603 Hz	1.92	-	1.0	1.0	1.0	1.0	1.0	1.0	1.0	1.0	*< 0.0001*
803 Hz	2.33	1.14	-	1.0	1.0	1.0	1.0	1.0	1.0	0.55	*< 0.0001*
1051 Hz	1.51	0.32	− 0.82	-	1.0	1.0	1.0	1.0	1.0	1.0	*< 0.0001*
1358 Hz	0.58	− 0.61	− 1.75	− 0.93	-	1.0	1.0	1.0	1.0	1.0	*< 0.0001*
1739 Hz	0.35	− 0.84	− 1.98	− 1.16	− 0.23	-	1.0	1.0	1.0	1.0	*< 0.0001*
2212 Hz	1.36	0.17	− 0.97	− 0.15	0.78	1.0	-	1.0	1.0	1.0	*< 0.0001*
2798 Hz	0.5	− 0.69	− 1.83	− 1.0	− 0.08	0.15	− 0.85	-	1.0	1.0	*< 0.0001*
3525 Hz	0.57	− 0.62	− 1.76	− 0.94	− 0.01	0.22	− 0.79	− 0.07	-	1.0	*< 0.0001*
4426 Hz	− 0.3	− 1.5	− 2.63	− 1.81	− 0.88	− 0.66	− 1.66	− 0.81	− 0.88	-	*< 0.0001*
5544 Hz	*− 8.42*	*− 9.61*	*− 10.75*	*− 9.92*	*− 9.0*	*− 8.77*	*− 9.77*	*− 8.92*	*− 8.99*	*− 8.11*	-

The degrees of freedom are 99. Combinations with a *p* value smaller 0.05 are indicated in italics

The spectral weight for ITDs at the lowest frequency band (442 Hz) was significantly higher than for all other bands (see Table 1). The weight at the 803 Hz band was found above the weights for the frequency bands from 1051 to 3525 Hz as well as for the 4426-Hz band. The weight for the 4426-Hz band was also found to be lower than for the highest band (5544 Hz). The highest significant spectral weight for ILDs (see Table 2) was at the highest frequency band (5544 Hz). No other significant differences were found for the spectral weights of ILDs when comparing across frequency bands. All weights with ITDs and ILDs were found to be larger than 0, indicated by the asterisks in Fig. 3, except for the weight at the 4426-Hz band with ITDs.

#### Effect of Uncorrelated Edge Bands

**Fig. 4:**
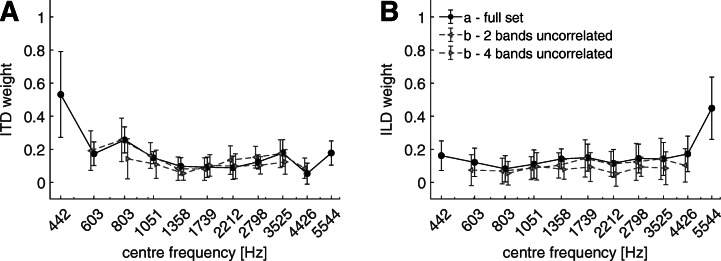
Spectral weights of ITDs (panel A) and ILDs (panel B) with respect to the center frequencies of the 1-ERB noise bands. The circles indicate the condition with interaural disparities on all eleven bands (full set), and the triangles indicate the condition with uncorrelated noise on the most outer frequency bands. The symbols and the error bars indicate the mean and the standard deviation for the ten listeners, respectively

Figure 4 shows the spectral weights for the reference conditions (experiment 1a, as in Fig. 3) and the weights for experiment 1b with interaurally uncorrelated noise bands at the spectral edges. The leftwards and rightwards pointing triangles indicate the conditions with two (the highest and the lowest) and four (the two highest and the two lowest) interaurally uncorrelated noise bands as edge bands, respectively. A linear mixed model of the ITD weights revealed that the frequency [*F*(10,234) = 31.88, *p* < 0.0001] and the conditions [*F*(2,234) = 6.57, *p* = 0.0017] were significant factors, while the interaction was not significant [*F*(14,234) = 0.96, *p* = 0.5]. The within factor analysis of the conditions revealed no significant difference between the reference condition and the condition with two uncorrelated edge bands [*t*(248) = − 1.04, *p* = 0.89] but a significant difference between the reference and the condition with four uncorrelated edge bands [*t*(248) = 2.62, *p* = 0.0277] was found. A linear mixed model of the ILD weights revealed that the factors frequency [*F*(10,234) = 19.98, *p* < 0.0001] and conditions [*F*(2,234) = 10.04, *p* < 0.0001] were significant, while the interaction was not significant [*F*(14,234) = 0.57, *p* = 0.89]. Similar to the ITD weights, the within factor analysis of the conditions with the ILD weights showed no significant difference between the reference condition and the condition with two edge bands with uncorrelated noise [*t*(248) = 2.37, *p* = 0.0556], but a significant difference to the condition with four edge bands with uncorrelated noise [*t*(248) = 4.52, *p* < 0.0001]. Since for both ITD and ILD weights no significant interaction effects were found, the shape with respect to frequency does not change. An offset with generally lower weights was however observed when presenting interaurally uncorrelated noise samples on the spectral edges.

#### Effect of Reduced Stimulus Bandwidth

**Fig. 5:**
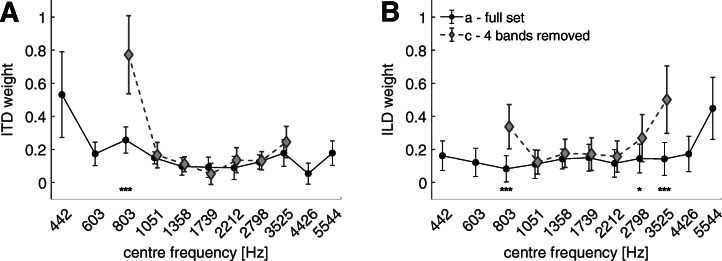
Spectral weights of ITDs (panel A) and ILDs (panel B) with respect to the center frequencies of the 1-ERB noise bands. The circles indicate the condition with interaural disparities on all eleven bands (full set), and the diamonds indicate the condition with removed stimuli on the two most outer frequency bands. The symbols and the error bars indicate the mean and the standard deviation for the ten listeners, respectively. Asterisks are used to indicate *p* values of weights relative to the full set condition. ***p***<***0.0001, **p***<***0.01, * *p****<***0.05, ·*p****<***0.1. If no asterisk is plotted below the weight, no significant difference to the full set condition was found

Figure 5 shows the spectral weights in the reference condition (experiment 1a, circle symbols) and the condition with removed edge bands (experiment 1c, diamond symbols). The linear mixed model of the ITD weights showed significant effects of both frequency [*F*(10,153) = 42.62, *p* < 0.0001] and condition [*F*(1,153) = 27.87, *p* < 0.0001] as well as of their interaction [*F*(6,153) = 18.21, *p* < 0.0001]. The multiple comparison analysis of the differences between the two conditions revealed that only a significant difference at the, in this condition, lowest frequency band (803 Hz) was found [*t*(153) = − 11.52, *p* < 0.0001]. For the ILD weights, the model showed significant effects of frequency [*F*(10,153) = 20.02, *p* < 0.0001], condition [*F*(1,153) = 68.92, *p* = 0.0001] and their interaction [*F*(6,153) = 12.34, *p* < 0.0001]. The effect of increased ILD weights was significant both at the lowest (803 Hz) [*t*(153) = − 6.61, *p* < 0.0001] and at the two highest frequency bands at 2798 Hz [*t*(153) = − 3.22, *p* = 0.0174] and 3525 Hz [*t*(153) = − 9.31, *p* < 0.0001].

### Experiment 2: Spectral Weights with Auditory Enhancement

**Fig. 6:**
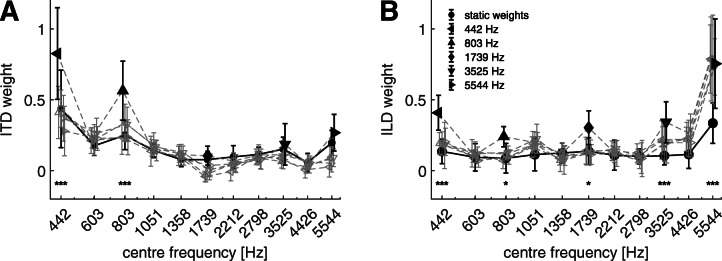
Spectral weights of ITDs (panel A) and ILDs (panel B) with respect to the center frequencies of the 1-ERB noise bands. The circles indicate the condition with interaural disparities on all eleven bands (experiment 1a). The gray lines and symbols indicate the conditions with enhanced bands, where the black symbol represents the on-frequency band. The symbols and the error bars indicate the mean and the standard deviation for the six listeners, respectively. Asterisks are used to indicate *p* values of weights relative to the full set condition. ***p***<***0.0001, **p***<***0.01, *p***<***0.05. If no asterisk is plotted below the weight, no significant difference to the full set condition was found

Figure 6 shows the spectral weights of ITDs (panel A, left) and ILDs (panel B, right) in the condition with pre- and post-cursors. The reference condition from experiment 1a is shown as circles, the off-frequency weights are indicated in grey and the on-frequency weights in black. The symbols at the bottom of the figures indicate the significance level between the reference condition and the on-frequency weight.

The linear mixed model of the ITD weights revealed significant effects of frequency [*F*(10,325) = 71.86, *p* < 0.0001] but not of condition [*F*(5,325) = 0.44, *p* = 0.82]. The interaction between the two main factors was significant [*F*(50,325) = 4.66, *p* < 0.0001]. The comparison of the on-frequency weights with the reference condition revealed significant increases for the 442-Hz band [*t*(325) = − 7.2, *p* < 0.0001] and the 803 Hz [*t*(325) = − 5.8, *p* < 0.0001] band but not for the 1739 Hz [*t*(325) = − 0.49, *p* = 1.0], 3525 Hz [*t*(325) = − 0.62, *p* = 1.0], and 5544 Hz [*t*(325) = − 1.31, *p* = 0.96] bands.

The analysis of the linear mixed model of the ILD weights showed a significant effects of the frequencies [*F*(10,325) = 102.63, *p* < 0.0001], conditions [*F*(5,325) = 8.43, *p* < 0.0001], and their interaction [*F*(50,325) = 2.77, *p* < 0.0001]. Comparing the on-frequency weights to the corresponding weights in the reference condition showed a significant increase for all tested frequencies. The largest increase was found for the lowest [*t*(325) = − 4.68, *p* < 0.0001] and the highest [*t*(325) = − 7.2, *p* < 0.0001] frequency band, but the 3525-Hz band [*t*(325) = − 4.06, *p* = 0.0003] also received a substantially larger weight when using the pre- and post-cursors. The 803-Hz [*t*(325) = − 2.61, *p* = 0.0469] and 1739-Hz [*t*(325) = − 2.86, *p* = 0.0228] bands were also found statistically different from the reference weights but with a smaller increase than the other on-frequency bands.

## **DISCUSSION**

In the present study, the spectral weighting for a stimulus consisting of 11 simultaneously presented 1-ERB-wide noise bands with ITDs or ILDs was investigated. It was shown that the highest weight for ITDs was given to the frequency band with the lowest center frequency, and the highest weight for ILD was given to the frequency band with the highest center frequency. The remaining bands received substantially lower weights than these edge bands. This “edge effect” was also found when reducing the overall bandwidth of the stimulus. When presenting interaurally uncorrelated noise as the edge bands, no change in weight was observed, resulting in a weighting function with equal spectral weights. The auditory enhancement paradigm in experiment 2 led to an increase of the on-frequency band. This enhancement of the weight was found for ITDs at low frequencies and for ILDs at low and high frequencies.

The results from this study are in general agreement with the Duplex theory (Rayleigh 1907; Macpherson and Middlebrooks 2002): ITDs receive the highest weight at low frequencies and ILDs at high frequencies. However, when assuming that ITD information is prominent at low frequencies and that the amount of useful information gradually decreases towards high frequencies (Klumpp and Eady 1956; Brughera et al. 2013), and vice versa for ILDs (Mills 1960), the findings of the present study show a different pattern. Instead of a gradual change of the weights, a sharp transition from high to low weights was found at the frequency bands located at the spectral edges of the stimulus. Such an edge effect has previously been shown in temporal weighting functions using a similar method (e.g., Stecker and Hafter 2002). Generally, the auditory system seems to weigh information on the edges stronger, as found for the binaural edge pitch effect (Klein and Hartmann 1981) or for loudness perception of multiple spectral or temporal components (Joshi et al. 2016; Oberfeld et al. 2012). However, this edge effect is at odds with estimates of spectral weights based on ITD and ILD thresholds where a gradual change of the spectral weight for ITDs has been shown (Raatgever 1980; Stern et al. 1988; Buchholz et al. 2018). A reason for this difference might be that the listeners in the current study had to integrate the binaural information across frequencies and, thus, binaural interference (McFadden and Pasanen 1976), i.e., across-channel interference, was taken into consideration.

Besides the possibility of additional effects being present when lateralizing broadband stimuli as in the present study compared with the isolated narrowband stimuli as used in previous studies, there might exist a fundamental difference in the applied methods. A direct comparison of these methods using identical stimuli would be required to rule out this systematic factor.

The current study was designed to reduce within-channel interference by separating the 1-ERB-wide noise bands by 1-ERB-wide spectral gaps. Thus, only little energy “leaked” into neighboring auditory channels. However, binaural auditory filters have been shown to be wider than monaural auditory filters (van de Par and Kohlrausch 1999; Kolarik and Culling 2010; van der Heijden and Trahiotis 1998; Bernstein and Oxenham 2006; Holube et al. 1998). Assuming wider binaural auditory filters, this might have introduced a within-channel interference of the interaural disparities. This possible interference might have led to a reduction of the weight in a given frequency band as conflicting binaural information from two or more bands were integrated within one binaural auditory filter. Thus, the frequency bands at the spectral edges with only a single neighboring band were less affected by within-channel interference than the remaining bands. This explanation is supported by the conditions with altered edge frequency bands where bands were removed or replaced with uncorrelated noise. When a band was removed, the interference for the new edge band is reduced and thus the spectral weight of this band increases. However, when there is uncorrelated noise on the edge band, the interference remains constant and thus the weight is unchanged. However, the edge effect only occurred if usable interaural information for the auditory system were available. This was the case mainly in low frequencies for ITDs and in high frequencies for ILDs. Yet, in the condition with removed edge bands, also the lowest frequency band for ILDs was increased in comparison with the reference condition, which suggests that enough ILD information was available at the mid-frequency range but not at the low-frequency range. An alternative explanation is that the ITD of 0 μs was a stronger opposing cue at the lower frequency band and thus the ILD weight was lower.

Because of the behavioral nature of the present study, other mechanisms might underlie the results. One alternative interpretation for the increased weights at the edge frequencies and for the conditions with imposed auditory enhancement (experiment2) could be a perceptual separation of one of the noise bands. In the light of this interpretation, if none of the noise bands would be perceptually separated, there is statistically seen a very small probability that the cue of one band would dominate the perception. Any attribute in the stimulus that would help to separate one noise band from the others might then result in the judgment of the listener to be dominated by the cue imposed on that specific band. Such a separation could happen due to spectral placement at the edge (see also Klein and Hartmann 1981), or by preceding or following sounds in the same spectral region (Viemeister 1980; Byrne et al. 2011). If no other mechanism leading to perceptual weighting existed, then all weights would be equally low in the absence of a cue supporting separation. The edge frequencies would then receive high weights as a consequence of perceptual separation (lowest for ITD due to the presence of phase locking, and ILD at high frequencies). This explanation would require that all bands are processed independently from each other and that the importance of the cues would be constant across frequency. The connection to phenomena like binaural interference and the gradual decrease of phase locking from low to high frequencies as well as a mutual interaction of spectral components in such listening conditions might provide some additional insights into the mechanisms underlying the behavioral data.

The mechanisms in the auditory system that might underlie a frequency weighting are not fully known. Previous studies have found specific sensitivity to spectral edges in the dorsal cochlear nucleus (DCN) (Reiss and Young 2005). While the DCN has been shown to play a role in sound localization both in azimuth and elevation and projects directly to the inferior colliculus (IC) (May 2000), it is bypassing binaural structures. Thus, it is not clear if the monaural DCN might affect binaural processing. One might also speculate that adaptation effects as observed at the level of the IC might lead to an enhancement of spectral edges due to the asymmetry in excitation around the edge band (Nelson and Young 2010). The perceptually measured frequency weighting might, however, be the compound result of such various phenomena along the auditory pathway. Hence, physiological studies will have to provide the insight into the exact origin of the observed weights.

Assuming that the ITD and ILD detection thresholds for the stimulus of the current study are similar to the thresholds proposed in the literature for narrow-band stimuli, a specific value of ITD or ILD could lead to differences in the amount of lateralization when presented in different frequency bands. Hence, one might interpret that a lower weight of ITD at higher frequencies is caused by the fact that it was close to or even below detection threshold compared with a lower frequency where the corresponding ITD was well above detection threshold. The data of the current study are partially in agreement with this interpretation. The weights are, however, even at the highest frequencies not equal to zero which indicates a small contribution of these cues, even at high frequencies. In order to quantify this in more detail, detection threshold and the role of the magnitude of the cue above detection threshold needs to be investigated with more complex stimuli such as those used in the present study.

Sensitivity to a specific stimulus attribute and the perceptual weights likely provide different information about sensory processing. It has, however, been argued that these two measures might be interconnected (Leibold et al. 2009; Kortekaas et al. 2003). This aspect has been discussed in the light of signal detection theory and, for example, the interaction between streaming and masking (Lutfi et al. 2012; Chang et al. 2016). In the current study, it is unclear if the detectability of, for example, ITD in frequency bands above 1.5 kHz might have an impact on the derived weights. It is challenging to compare weights derived from sensitivity for isolated pure tones (Brughera et al. 2013) with those of narrowband noises (Buchholz et al. 2018), as sensitivity for these stimuli differs. In order to shed light on these factors, a comparison across these data might be possible when considering these different stimuli in conditions with a constant *d′* and to evaluate the performance in the signal detection theory framework outlined in Lutfi et al. (2012). Such a framework would then allow to include the detectability of each cue in each frequency by a combination of *d′*. The results of the present study and of the previous studies provide a good starting point to extend the current point of view in this direction. The paradigm applied in the present study allows to evaluate the overall performance even in the presence of the many possible parameter combinations, at the cost that detailed information on the interactions might be obscured.

Multiple studies showed that spectral bandwidth plays a role in processing of binaural information, but in a non-trivial way. Thavam and Dietz (2019) directly compared ITD thresholds for pure tones, tone complexes, and noises of different bandwidth and spectral shape. In their data, a lower ITD threshold was found for a white noise filtered between 600 and 1000 Hz than for pure tones or tone complexes. The ITD threshold was similarly low, however, for a 20- to 1400-Hz noise. Hence, congruent information across frequency ranges exceeding one auditory filter might be beneficial in the processing of ITD, while incongruent information, as in binaural interference experiments, might be detrimental. These results suggest that the weights derived in the present study might differ for other stimulus parameters, spectral shape, and salience of the provided cues.

In experiment 2, spectral weights were determined while using an auditory enhancement paradigm (Viemeister 1980; Byrne et al. 2011). The weights at the cued bands were found to be increased at low and high frequencies for ILDs and at low frequencies for ITDs with respect to the reference condition. The results are in line with findings on ITD and ILD detection thresholds as discussed above. Comparing the weights across the enhanced bands, it is apparent that the edge frequency bands at low and high frequencies receive the highest weights for ITDs and ILDs, respectively. This is not in line with threshold measurements of ITDs and ILDs. While ILD thresholds are constant over frequency except at around 1 kHz (Yost and Dye 1988; Grantham 1984; Rowland and Tobias 1967), ITD thresholds have been shown to be lowest at around 800 Hz (Klumpp and Eady 1956; Brughera et al. 2013).

The reason for the change of the spectral weights using the auditory enhancement paradigm remains unclear. One possibility could be due to an internal gain of the enhanced band. This might lead to an increase in loudness of that band (Viemeister 1980) or to an otherwise perceptually separated band. Second, both the enhancement and the binaural processing might happen at the same stage of the auditory system, which might lead to a more efficient coding of information in the enhanced band. The underlying mechanism in the auditory system for the auditory enhancement effect is unclear (Feng et al. 2018; Beim et al. 2015). Some studies suggested the auditory nerve as the origin of the auditory enhancement (Summerfield et al. 1987; Palmer et al. 1995); in other studies, higher stages such as the inferior colliculus (Nelson and Young 2010; Feng et al. 2018) or the auditory cortex (Feng et al. 2018; Carcagno et al. 2014) were suggested as the possible origins. Carcagno et al. (2013) stated that the auditory enhancement likely occurs at a stage of the auditory system where the monaural auditory pathways have converged, which is in agreement of the findings in the current study where the auditory enhancement paradigm leads to increased weights. Third, the reason for increased weights could be due to a reduced binaural interference. Best et al. (2007), and Woods and Colburn (1992) showed that binaural interference is reduced when auditory information is grouped. However, grouping has been argued to not be a reason for auditory enhancement (Summerfield et al. 1987; Byrne et al. 2011); thus, it might also not be the underlying mechanism for the increased spectral weights. The current study can neither prove nor rule out any of the three reasons and further work is needed to link auditory enhancement and binaural perception.

Previous studies have shown that ITD information carried by the temporal fine structure (TFS) of the signal can be used by the auditory system up to about 1500 Hz, while envelope ITD information can also be used at higher frequencies. In the present study, listeners seemed to primarily rely on low-frequency TFS information when judging the lateralization with ITD cues. This is in line with findings showing that TFS cues are weighted higher than envelope cues (Moore et al. 2018). Additionally, when using the enhancement paradigm, listeners only gave a higher weighting to the low-frequency bands with TFS information but not to the high-frequency bands where only envelope information is available.

In the current study, the spectral weighting of ITDs and ILDs has been investigated in separation. However, to lateralize a sound, both ITDs and ILDs are used jointly. Thus, one might investigate a common lateralization weighting function.

Even though the edge frequency bands have been found to receive the highest weight, also the other weights were found to be above zero. Thus, all frequencies were found to contribute to the lateralization.

## **CONCLUSIONS**

In the current study, we investigated the across-frequency integration of interaural time and level differences. An observer-weighting analysis paradigm was used where spectral weights of ITDs or ILDs were determined using a multiple linear regression approach. It has been shown that ITD weights are largest at the lowest frequency band and ILD weights are largest at the highest frequency band. When using an auditory enhancement paradigm, these weights increase at the enhanced frequency band while the remaining frequencies remain constant. Thus, bands that are perceptually separated from the remaining stimulus receive a higher apparent weight.
